# Defining targets to improve care delivery for T4 larynx squamous cell carcinoma

**DOI:** 10.1002/lio2.959

**Published:** 2022-11-16

**Authors:** Catherine H. Frenkel, Daniel S. Brickman, Sally J. Trufan, Matthew C. Ward, Benjamin J. Moeller, Daniel R. Carrizosa, Ashley L. Sumrall, Zvonimir L. Milas

**Affiliations:** ^1^ Division of Head and Neck Surgical Oncology, Department of Surgery Levine Cancer Institute, Atrium Health Charlotte North Carolina USA; ^2^ Department of Biostatistics Levine Cancer Institute, Atrium Health Charlotte North Carolina USA; ^3^ Department of Radiation Oncology Levine Cancer Institute, Atrium Health Charlotte North Carolina USA; ^4^ Department of Medical Oncology Levine Cancer Institute, Atrium Health Charlotte North Carolina USA

**Keywords:** laryngectomy, larynx cancer, NCDB

## Abstract

**Objective:**

United States oncology trends consistently demonstrate that nearly half of T4a larynx carcinoma patients are treated with larynx preservation, despite national guidelines favoring laryngectomy. This study identifies clinical decision‐making drivers and defines patient subsets that should become targets for care improvement.

**Methods:**

Retrospective analysis of patients with cT4 squamous cell carcinoma of the larynx from US National Cancer Database 2005–2016. Demographic data and survival rates between clinical pathways were compared. Survival was estimated by Kaplan–Meier method with statistical comparisons assessed by log‐rank test.

**Results:**

Of 11,556 patients with cT4 disease, laryngectomy (TL) was the initial treatment for 4627 (40%) patients. Larynx preservation via chemoradiation (CRT) occurred for 4307 patients. TL and CRT patients had similar Charlson–Deyo comorbidity indices and insurance status. TL patients had higher total tumor size, lower N3 rates and were more often seen at academic institutions (*p* < .0001). N0 surgery patients with adjuvant treatment demonstrated superior median survival (MS) compared to CRT (surgery + radiation MS: 69 months, surgery + chemoradiation MS: 66, CRT MS: 37.7), *p* < .0001. MS for N1/N2 disease patients was 56.5 months for surgery + radiation and 35.5 months for surgery + CRT, superior to CRT, MS 30.8 months, *p* < .0001. Tri‐modality N3 patients with up front surgery had similar MS compared to CRT (surgery + chemoradiation 21.3 months vs. CRT 16.1), *p* = .95.

**Conclusion:**

National quality improvement initiatives are needed to promote guideline adherence and improve survival in advanced larynx cancer. Targets for such initiatives should be patients with limited or no nodal disease burden, that meet clear T4a imaging criteria.

**Level of Evidence:**

Level IV, non‐randomized controlled cohort.

## Graphical abstract

Retrospective analysis of patients with cT4 squamous cell carcinoma of the larynx from US National Cancer Database 2005–2016. This study identifies clinical decision‐making drivers and defines patient subsets that should become targets for advanced larynx cancer care improvement.

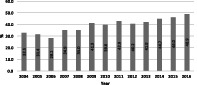

## 1 INTRODUCTION

National guidelines and expert consensus support the management of most resectable T4 larynx cancers with laryngectomy.^1^ However, US surgical trends consistently demonstrate that the minority of patients with T4a larynx carcinoma undergo primary laryngectomy.^2^ The limitations in delivering the standard of care intervention likely stems from the challenges of defining quality‐centric care in advanced larynx cancer. The drivers of care quality for T4 larynx cancer patients are complex. The multidisciplinary team caring for T4 larynx cancer patients must consider laryngectomy‐free survival, rates of salvage laryngectomy, overall survival, rates of triple therapy, individual patient needs, and tumor‐specific characteristics.

Recent published analyses of T4 larynx cancer emphasize survival, but avoid considering patient or tumor‐specific characteristics, rates of triple therapy and larynx preservation.^3^, ^4^, ^5^, ^6^ Whether laryngeal preservation (LP) in resectable T4 larynx cancer compromises survival is also a subject of international debate.^7^, ^8^, ^9^, ^10^ Additionally, the decision for or against laryngectomy is not binary. Some institutions gauge tumor response to systemic therapy, predicting the patients likely to succeed in LP and those who should pivot to laryngectomy.^11^, ^12^ The purpose of this study is to examine current practice patterns with respect to larynx preservation, salvage surgery, and survival outcomes for T4 larynx cancers using the U.S. National Cancer Database. Patient subgroups that would most benefit from quality of care improvements in survival and satisfaction will be identified.

## 2 MATERIALS AND METHODS

This retrospective cohort analysis utilized data from the National Cancer Database (NCDB), a clinical oncology registry with data from more than 1500 Commission on Cancer‐accredited facilities.^13^ The database is jointly sponsored by the American College of Surgeons and the American Cancer Society. It represents more than 70 percent of newly diagnosed cancers nationwide. This study was deemed exempt from institutional review board approval. Patients with larynx squamous cell carcinoma were identified by site‐specific (Larynx, C32) surgery of primary site codes (40, 41, 42, 50, 80) and International Classification of Diseases for Oncology—3 histologic codes (8052‐78, 83‐4). Only data associated with clinical T4 patients without distant metastases (M0) were analyzed. Surgery first patients were defined as any patient undergoing total or radical laryngectomy or pharyngolaryngectomy, either alone or prior to radiation and/or chemotherapy. Of those patients undergoing primary total laryngectomy (TL), adjuvant treatment was defined as radiation, at least 40 Gy, delivered within 90 days of surgery, with or without chemotherapy.^2^ Patients who did not undergo laryngectomy first were divided into the following subgroups—chemoradiation (CRT) and other. CRT was limited to patients undergoing treatment within 90 days of diagnosis, who received at least 50 Gy of radiation.^2^ Data regarding chemotherapeutic agents and duration or completion of chemotherapy courses are not available in the NCDB. Therefore, chemotherapy was treated as a binary variable, with any or no chemotherapy delivered. Standard of care/intent to cure pathways were surgery (TL) plus adjuvant radiation with (SRC) or without (SRT) chemotherapy, or CRT. Patient demographics, treatment, and tumor characteristics were identified. Chi‐squares tests were used to compare descriptive and demographic data. Participation in a standard of care surgical pathway with adjuvant treatment (as defined above) or nonsurgical pathway (as defined above), median survival (MS) and 2‐year survival (2YS) were evaluated. Survival was estimated by the Kaplan–Meier method and the significance of difference among the curves was calculated by the log‐rank test. All analyses were performed using SAS 9.4 (SAS Institute Inc., Cary, NC). Statistical significance was set at .05.

## 3 RESULTS

**FIGURE 1:**
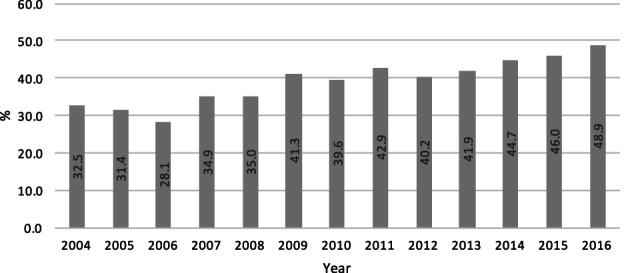
Increasing percent of cT4 larynx cancers managed by laryngectomy over time

We identified 11,556 patients ≥18 years diagnosed between 2004 and 2016 with clinical T4 M0 larynx squamous cell carcinoma. Survival data was available for 10,531 patients. Laryngectomy was the initial treatment for 4627 (40%) patients, 89% of whom had survival data (*n* = 4126). Annual rates of laryngectomy in cT4 patients ranged from 28.1% to 48.9% of total cases, with a trend toward TL over time (*p* < .0001) (Figure 1).

Of the 6929 patients who did not receive TL, survival data was available for 6405 patients (92.4%). Clinical T4a disease was documented in 93.2% of LP patients (*n* = 6161), cT4b disease was documented in 1.6% of TL patients (*n* = 68), and 600 patients were classified as cT4 NOS. In the no surgery first group, 4307 patients initiated chemoradiation.

Laryngectomy versus no laryngectomy (*n* = 6929) patients were of similar age and racial distribution (Table 2). They had similar Charlson–Deyo comorbidity indices and rates of having medical insurance. However, fewer females and patients outside of academic institutions received TL (*p* < .0001). TL patients had higher T4a and N0 rates, lower N3 rates, and less frequent poorly differentiated histology (*p* < .0001). Laryngectomy patients also had a significantly greater total tumor size (*p* < .0001, mean 91 ± 45 mm, median, IQR 83 mm, 43 mm vs. 43 ± 38 mm, median, IQR 36 mm, 32 mm). Site‐specific features of the primary tumor are described in Table 2.

Pathologic features of the resected tumors included a 10.1% rate of being down T‐staged after surgery to pT3 (*n* = 460). Pathologic N0 status was noted in 41.4% of patients (*n* = 1914), versus 52.3% of patients with a pre‐operative cN0 status (*n* = 2414). One lymph node was positive for metastatic carcinoma for 708 patients, 2 nodes were involved for 424 patients, and 3 or more lymph nodes had evidence of disease in 1133 patients. Nodal size >6 cm (1.1%) correlated with clinical N3 status (1%). Most nodes were 3 cm or smaller (74.6%). The positive margin rate was 13.6% (*n* = 631). Pathologic extranodal extension was present in 970 patients.

The entire T4 cohort demonstrated a MS of 26.6 months and 2YS of 52.6%. Patients embarking on a standard treatment pathway, predictably, survived longer (surgery 1st MS 45.1 months, CRT MS 34.1 months, *p* < .0001). Limiting the data set to T4a patients had little effect on survival (Table 1). Median survival of CRT patients requiring salvage (MS 33.3 months) was most similar to that of the CRT.

**TABLE 1:** Survival of cT4 larynx squamous cell carcinoma based on treatment pathway

	*n*	Event	Censored (%)	MS (months)	95% CI	2YS (%)	95% CI
**All T4**	10,530	6892	6892 (65)	26.6	25.6–27.8	52.6	51.6–53.6
**Surgery 1st**	4126	2320	2320 (56)	45.1	42.0–47.5	65.4	63.9–66.9
T4a	3789	2126	1663 (44)	45.1	42.0–47.6	65.6	64.0–67.1
Negative margin	3478	1864	1864 (46)	50.7	46.9–54.7	68.3	66.7–69.9
Positive margin	564	405	159 (28)	21.8	19.6–25.1	47.5	43.2–51.6
N0	2151	1136	1015 (47)	56.0	51.8–60.4	71.3	69.2–73.2
N1–N2	1871	1116	755 (40)	33.4	30.6–36.6	59.1	56.8–61.4
N3	39	32	7 (18)	20.1	11.1–53.9	43.4	27.7–58.2
**Surgery alone**	1218	756	462 (38)	34.7	31.2–38.6	59.5	56.6–62.3
T4a	1115	692	423 (38)	34.7	31.0–38.6	59.2	56.2–62.2
Negative margin	1060	628	432 (41	40.1	35.4–45.5	63.1	60.0–66.0
Positive margin	141	116	25 (18)	13.2	10.1–18.2	36.1	28.0–44.2
N0	744	441	303 (41)	43.3	37.4–48.6	65.6	61.9–69.0
N1–N2	439	292	147 (33)	23.3	18.1–27.1	49.6	44.7–54.3
N3	12	11	1 (8)	11.2	4.4–55.4	33.3	10.3–58.8
**Surgery** **+** **radiati**n	1073	512	561 (52)	63.6	57.5–73.5	75.0	72.2–77.6
T4a	995	473	522 (52)	63.4	57.5–72.9	75.4	72.5–78.1
Negative margin	959	447	512 (53)	65.9	60.4–76.5	76.1	73.2–78.8
Positive margin	101	59	42 (42)	40.7	32.7–57.7	67.0	56.5–75.6
N0	629	293	336 (53)	69.0	60.6–81.4	78.7	75.1–81.8
N1–N2	429	210	219 (51)	56.5	44.1–66.7	69.9	65.1–74.2
N3	2	1	1 (50)	101.4	NA	100.0	NA
**Surgery + radiation + chemotherapy**	975	537	438 (45)	44.7	39.7–49.6	65.7	62.5–68.7
T4a	896	497	399 (45)	44.7	39.4–49.4	65.7	62.4–68.8
Negative margin	737	383	352 (48)	49.7	44.0–65.0	68.3	64.7–71.7
Positive margin	201	134	67 (33)	26.4	21.1–35.5	53.4	46.0–60.2
N0	356	172	184 (52)	66.0	48.9–74.8	74.2	69.1–78.6
N1–N2	587	345	242 (41)	35.5	29.9–41.9	61.1	56.9–65.0
N3	17	13	4 (24)	21.3	11.1–53.9	47.1	23.0–68.0
**No surgery 1st**	6404		4572 (71)	19.2	18.1–20.1	44.3	43.1–45.6
N0	2551	1789	762 (30)	21.4	19.6–23.4	47.4	45.4–49.4
N1–N2	3427	2448	979 (29)	18.9	17.6–20.2	43.4	41.7–45.1
N3	318	258	60 (19)	12.0	9.7–13.5	29.8	24.7–35.1
**Radiation + chemotherapy**	3153	1983	1170 (37)	34.1	32.4–36.9	59.8	58.0–61.6
T4a	2401	1515	886 (37)	34.0	31.7–36.8	59.6	57.6–61.6
N0	1045	653	392 (38)	37.7	33.8–43.2	63.2	60.1–66.1
N1–N2	1488	969	519 (35)	30.8	27.2–33.7	56.4	53.7–58.9
N3	92	66	26 (28)	16.1	12.5–26.9	42.3	31.8–52.5
**Radiation + chemotherapy + salvage** +	161	97	64 (40)	33.3	28.5–44.9	61.9	53.6–69.1
N0	70	41	29 (41)	47.3	31.2–70.8	66.6	53.8–76.7
N1–N2	86	53	33 (38)	30.9	22.5–37.2	56.9	45.3–66.9
N3	3	3	0 (0)	32.8	13.5–61.3	66.7	5.4–94.5

Abbreviations: MS, median survival; YS, year survival.

**FIGURE 2:**
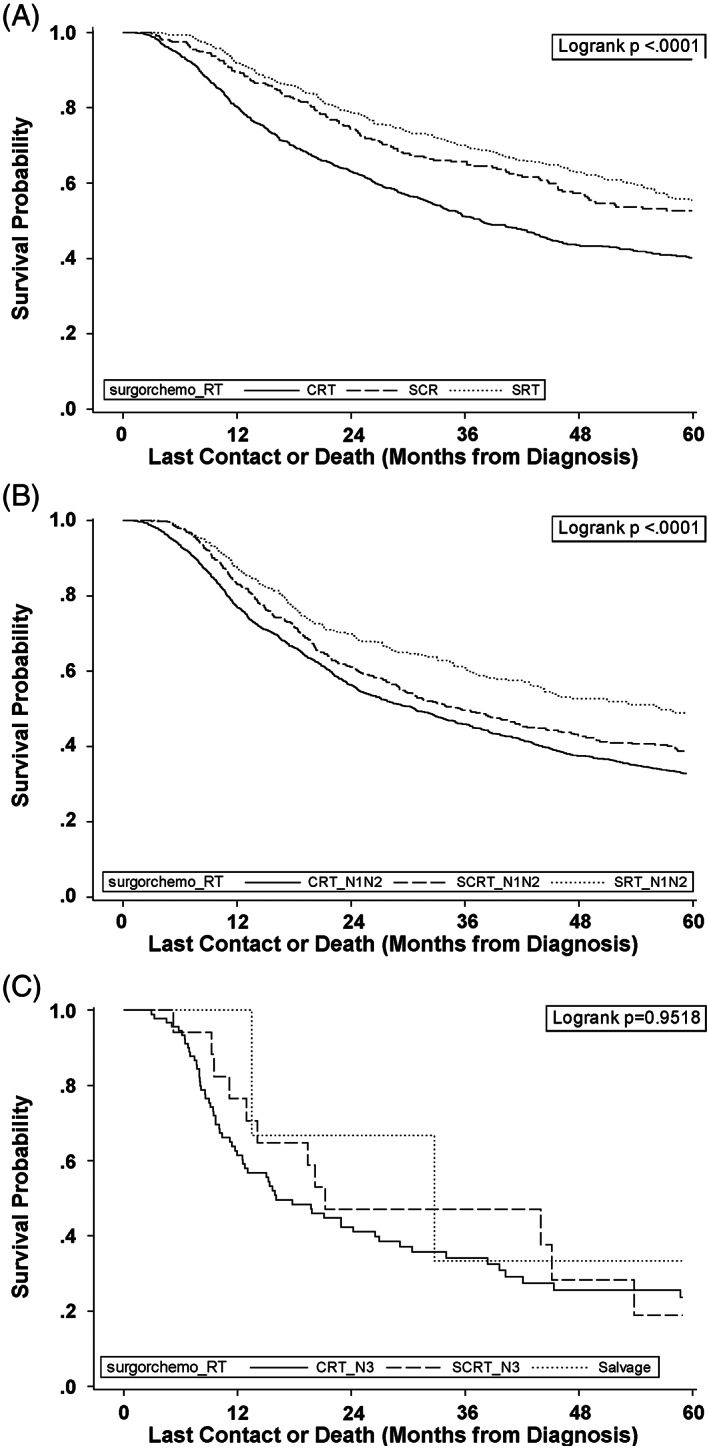
Kaplan–Meier survival based on treatment pathway and nodal status. Kaplan–Meier survival curves for patients with (A) N0, (B) N1 and N2, or (C) N3 treated with chemoradiation therapy (CRT), surgery with chemoradiation therapy (SCRT), surgery with radiation therapy (SRT), or salvage. *p*‐Values denoted on each graph. (*N* = 11,556)

cN0 patients represent 45% of the T4 larynx cancer population. SRT patients with N0 disease demonstrated similar MS with or without chemotherapy (Table 1). Survival of N0 CRT patients was inferior to that of surgical patients, MS 37.7 months. N0 CRT patients demonstrated worse survival outcomes than surgery with any or no adjuvant treatment (Figure 2A). Laryngectomy with adjuvant RT in node negative patients conferred the best calculated survival of all the analyzed patient subpopulations, with approximately an 80% 2‐YS and over a 50% survival rate at 5 years.

cN1‐2 patients represent 51% of the T4 larynx cancer population. N1/2 patients had similar rates of surgery alone (*n* = 439), SRT (*n* = 429) and SRC (*n* = 587). The best MS was exhibited by the SRT group, 56.5 months. MS of SRC patients was 35.5 months and was superior to that of CRT (MS 30.8 months) (Figure 2B; *p* < .001). Whereas the CRT cohort contained a greater proportion of T4b patients (23.9% vs. 8.1%), the SRC group had a positive margin rate of 20.6%. The total number of SRC patients, *n* = 975, was expectedly similar to the incidence of pathologic extranodal extension (*n* = 1062).

The number of N3 patients undergoing laryngectomy 1st was limited (*n* = 39). The majority of N3 patients did not undergo laryngectomy first (*n* = 318). Thirteen of the 14 N3 surgical patients (93%) with adjuvant therapy and survival data received triple therapy. Ninety‐two patients had CRT. Tri‐modality therapy patients with up front surgery had no significant difference in MS compared to CRT (SRC MS 21.3 vs. CRT MS 16.1; *p* = .95). (Figure 2C). Few N3 patients undergoing CRT underwent salvage surgery (*n* = 3). MS for this subgroup was 32.8 months (95% CI 13.5–61.3).

## 4 DISCUSSION

This study corroborates previous data which demonstrate that approximately half of resectable T4 larynx cancer patients do not undergo laryngectomy first.^2^, ^3^ Figure 1 interestingly identifies laryngectomy rates were at their nadir in 2006, with a gradual increase over time. The reason for this trend is likely multifactorial. However, it could be associated with the clinical impact of the American Society of Clinical Oncology's publication of their practice guidelines for the use of Larynx‐Preservation Strategies in 2006.^14^

Guidelines favor laryngectomy for patients with cartilage or neck soft tissue invasion unless the patient declines. The data behind the NCCN guidelines, and the limitations of this data are well‐known. The VA Larynx trial demonstrated a low rate of successful CRT without salvage laryngectomy in T4 patients. However, the trial was of limited applicability to T4 patients, as three‐quarters of this cohort had less bulky disease at the primary site.^15^ Subsequent RTOG 91‐11 LP data was also of limited applicability to T4 patients. Less than 10% of patients were cT4 and the study excluded bulky T4 disease.^16^ Given the limited data from randomized control trials to support LP for T4 larynx cancer, management recommendations have been guided by historical standards of care and multivariable regression analyses of national data.^2^, ^3^, ^17^ This study is unique from previous national data analyses, in that it uses the outcomes data to explore the clinical drivers impacting decisions between laryngectomy and larynx preservation for T4 patients, in order to direct future initiatives to deliver improved quality‐based care.

Practice patterns may diverge from NCCN guidelines because the guidelines fail to honor the complexity of clinical decision‐making in a patient‐centric system. The UK guidelines allow providers to consider LP for T4a tumors based on patient and tumor characteristics.^10^ Flexible guidelines enhance the ability of providers to offer patient‐centric recommendations that optimize outcomes and quality of life. The data from this study suggests a lack of consensus (either patient or provider driven) as to whether LP universally achieves inferior outcomes in all T4 patients given their clinical heterogeneity. For example, in this study management paradigms favor laryngeal preservation in the setting of low volume T4a disease. Clinical staging has inherent limitations that result in over and under‐staging, which would translate to management recommendation discrepancies if this could be known preoperatively. T4a patients who do proceed to laryngectomy have a 10.1% probability of being down T‐staged to pT3, for which the ideal management may have been laryngeal preservation. The positive margin rate was 13.6%, suggesting some resected patients may have unanticipated T4b disease. No clinical staging system is perfect. The degree of thyroid cartilage invasion, carotid encasement or prevertebral involvement cannot always be anticipated by staging imaging. Of course, inaccuracies in clinical staging will not be captured in the larynx preservation group. However, a strength of this study is that it acknowledges limitations in accuracy of clinical staging. Despite this, the likelihood of successful larynx preservation must be weighed against the value of an initial laryngectomy.

It may be reasonable for providers to balance survival and laryngectomy in N1‐2 patients with suspected extranodal extension against the probability of a nonfunctional larynx or persistent disease after CRT. In this study, the gap between survival for N1/N2 surgical patients requiring triple therapy and CRT alone is narrow (SRC vs. CRT MS of 35.5 vs. 30.8 months). Al‐Mamgani et al. voices a similar conclusion based on comparison of a random cohort of T4 larynx/hypopharynx squamous cell carcinomas with primarily N1‐2 disease (61%) undergoing LP to a historical surgical cohort.^5^ The study concluded that, while an overall survival benefit to surgery was unclear, ENE and a dysfunctional larynx were clearly associated with inferior survival. Most SRC patients exhibit a combination of adverse survival risk factors, primarily the addition of ENE and also possibly the risks associated with systemic therapy.^18^ These survival data may indicate a failure of tri‐modality therapy to overcome the negative prognostic value of ENE, indicating further research is needed into intensification.

The N3 patient subset has poor overall survival regardless of treatment pathway and most patients in this cohort did not have a laryngectomy first (*n* = 318 vs. SRC *n* = 39). This study found no significant benefit in long‐term survival for N3 patients who receive SRC versus CRT. These findings are echoed by Harris et al.^10^ Harris et al. reviewed survival in advanced stage larynx cancer in SEER from 2004 to 2012.^10^ Overall survival and DSS survival were not improved for patients with N3 disease treated with surgery versus chemoradiation. The N3 cohort is unlikely to derive benefit from quality initiatives that change practice patterns, until medical advances can confer an additional survival benefit.

N0 status is the most common single N stage presentation of the T4 larynx cancer patient. In N0 patients, MS and 5‐year survival rates are better after laryngectomy than CRT. This is regardless of whether the patient has no adjuvant therapy, a positive margin and radiation alone, or SRC. Only 10% of N0 patients demonstrated occult nodal metastases on final pathology. If a patient has surgically resectable T4a N0 disease and is a safe operative candidate, this study, as have others, support that the patient should be referred to a qualified surgeon to be counseled regarding laryngectomy.^3^, ^10^, ^17^ A significant survival disparity exists between LP and laryngectomy treatment pathways for this N0 patient subset. Quality improvement initiatives to promote guideline adherence and improve survival in larynx cancer are likely to have the greatest impact for this population.

The findings of this study demonstrate that organ preservation is more commonly practiced by nonacademic institutions. Chen et al. observed that patient survival for nonsurgical organ preservation treatment pathways at low‐volume facilities is significantly inferior.^17^ Such data calls to question whether LP at lower volume centers is a result of impaired access to a qualified surgeon. Patients with T4a larynx cancer should be encouraged to seek or obtain access to a surgical oncologist with a nuanced understanding of tumor characteristics, larynx function, and of patient characteristics.

Shortcomings of this study include the limitations inherent in a study based on administrative data. These include coding and data entry errors and incomplete data. Disease‐specific survival cannot be assessed in the NCDB. Histologic grade was analyzed, but histologic subtypes, some of which may behave more aggressively, were not considered. Subsites were not considered, but T4 cancers often involve the supraglottis, glottis and/or the subglottis concurrently. Detailed pathologic data beyond that which is reported by the NCDB's primary site‐specific tumor description (Table 2) is not known. The retrospective nature of this study limits interpretation due to selection bias, and is classified as level IV evidence. This is particularly relevant to the T4 laryngectomy population, in which patient health literacy, follow‐up and adherence to adjuvant recommendations can be unreliable. In our analysis, selection bias related to the CRT group was multidirectional. Nonsurgical patients were more likely to have smaller primary tumors or advanced, non‐resectable disease. Poor compliance with chemotherapy and or radiation treatment is likely to have impacted operative and nonoperative arms equally. Lin et al. reveals that CRT patients in the United States infrequently (24%) complete standard therapy.^19^

**TABLE 2:** Demographics and clinical characteristics of cT4 larynx squamous cell carcinoma patients

	Total population	Surgery as initial treatment		*p*‐Value
Variable	(*N* = 11,556)	Yes	No	Chi‐square
Age, years (mean ± SD)	61.9 ± 10.8	61.0 ± 10.1	62.4 ± 11.1	–
Sex, no. (%)				**<.0001**
Male	9335	3824 (82.7)	5511 (79.5)	
Female	2221	803 (17.4)	1418 (20.5)	
Race, no. (%)				.420
Caucasian	8891	3613 (78.1)	5278 (76.2)	
African American	2276	859 (18.6)	1417 (20.5)	
Other	389	155 (3.4)	234 (3.4)	
Institution type, no. (%)				**<.0001**
Academic	6063	3053 (66.0)	3010 (43.7)	
Other	5493	1574 (34.1)	3919 (56.6)	
Distance from hospital (miles), mean ± SD, median	33.0 ± 90.9, 12.1	42.0 ± 97.7, 17.7	27.0 ± 85.5, 9.6	–
Insurance, no. (%)				**.009**
Yes	10,117	4096 (88.5)	6021 (86.9)	
No	1439	531 (11.5)	908 (13.1)	
Charlson–Deyo comorbidity score, no. (%)				.670
0 or 1	10,360	4155 (89.8)	6025 (89.3)	
≥2	1196	472 (10.2)	724 (10.7)	
Tumor size (mm), no. (%)				**<.0001**
1–26	1492	719 (16.6)	773 (22.5)	
27–34	1499	830 (19.1)	669 (19.4)	
35–44	2111	1239 (28.6)	872 (25.3)	
≥45	2676	1547 (35.7)	1129 (32.8)	
Clinical T stage, no. (%)				**<.0001**
4 NOS	600	281 (6.1)	319 (4.6)	
4A	10,439	4278 (92.4)	6161 (88.9)	
4B	517	68 (1.5)	449 (6.5)	
Clinical N stage, no. (%)				**<.0001**
0	5153	2414 (52.3)	2739 (39.6)	
1 or 2	5846	2100 (45.5)	3746 (54.2)	
3	381	48 (1.0)	333 (4.8)	
X	154	58 (1.3)	96 (1.4)	
Grade of differentiation, no. (%)				**<.0001**
Moderate or well	6899	3221 (69.6)	3678 (53.1)	
Poor	1938	1215 (26.3)	1504 (21.7)	
Other	2719	191 (4.1)	1747 (25.2)	
Primary site‐specific tumor description, no. (%)				**<.0001**
Deep base of the tongue	37	9 (0.4)	38 (1.0)	
Esophagus, oropharynx, neck soft tissue, outer thyroid cortex, thyroid gland involvement	4639	1960 (86.6)	2679 (72.8)	
Extension through/to strap muscles or skin	427	141 (6.2)	286 (7.8)	
Extension through/to deep extrinsic muscles of tongue, trachea	463	109 (2.4)	354 (9.6)	
Carotid encasement or contiguous with mediastinal structures, the prevertebral space	366	45 (2.0)	321 (8.7)	
Extracapsular extension, no. (%)				**<.0001**
Clinical	347	32 (3.2)	315 (77.4)	
Pathologic	1062	970 (96.8)	92 (22.6)	
Size of largest lymph node (cm), no. (%)				**<.0001**
≤3	7634	3451 (74.6)	4143 (60.1)	
>3–6	632	304 (6.6)	328 (4.8)	
>6	157	51 (1.1)	106 (1.5)	
Unknown	3133	821 (17.7)	2312 (33.6)	

Abbreviation: SD, standard deviation.

## 5 CONCLUSION

The ideal targets for quality improvement initiatives to promote guideline adherence and improve survival in advanced larynx cancer are patients with no to little nodal disease burden who meet clear T4a imaging criteria. The barriers to evidence‐based management of patients who meet these criteria should be the subject of future study. They could be related to patient health literacy or institutional surgical access.

## CONFLICT OF INTEREST

The authors have no relevant conflicts of interest to disclose.
